# CHILDREN’S HEALTH: Methylation Links Prenatal PAH Exposure to Asthma

**DOI:** 10.1289/ehp.117-a195

**Published:** 2009-05

**Authors:** Adrian Burton

Research suggests that a mother’s exposure to pollution during pregnancy may predispose her child to asthma, and there is preliminary evidence implicating trans-placental exposure to polycyclic aromatic hydrocarbons (PAHs)—generated mainly by the burning of fossil fuels and abundant in high-traffic areas. Until recently, progress in the study of prenatal exposures to PAHs and other pollutants has been hampered by a paucity of biomarkers for predicting asthmatic risk. Researchers from the University of Cincinnati and Columbia University Mailman School of Public Health now report that methylation of *ACSL3*, a gene expressed in lung and thymus tissue, may provide a possible biomarker linking prenatal exposure to PAHs to childhood asthma.

The researchers hypothesize that trans-placental exposure to PAHs might lead to aberrant DNA methylation changes, which in turn alter the expression of genes in the fetal lung or immune system, perhaps setting the stage for childhood asthma. When toxicant-induced aberrations in DNA methylation occur during critical developmental periods, there is evidence they can lead to inappropriate gene expression and disease later on.

“It’s thought that pollutants that can cross the placenta may alter the function of some systems in later life,” explains study coleader Shuk-mei Ho, director of the Center for Environmental Genetics at the University of Cincinnati. These prenatal exposures can predispose the fetus to develop compensatory—and sometimes detrimental—responses in anticipation of continued exposure after birth. “During fetal life, anticipatory responses to the future external environment may come about via changes in the course of tissue or cell differentiation, often via epigenetic reprogramming of genes by DNA methylation,” she says. “Our data support the concept that environmental exposures can interact with genes during key developmental periods to trigger disease onset later in life, and that tissues are being reprogrammed to become abnormal later.”

The researchers initially examined PAH exposure data for 729 female participants in a longitudinal cohort study, all of whom were living in high-traffic areas of Northern Manhattan and the South Bronx, where childhood asthma rates hover around 25%. Next they identified 10 women with PAH exposure above the group median PAH level of 2.3 ng/m^3^ (as determined by personal air monitoring) and 10 women with exposure below the median. Using methylation-sensitive restriction fingerprinting to examine the DNA of umbilical cord white blood cells collected from the women’s children at birth, the researchers identified gene sequences whose methylation status differed between the high and low maternal exposure groups. These included sequences in or near the promoter region of six genes expressed in the lung and/or lymphoid tissue that are involved in inflammation or other immune response functions.

When the researchers compared the methylation percentages of these six gene sequences with their transcript expression levels in matched fetal placental tissue, the strongest inverse relationship (due to methylation’s silencing of the gene) was seen for *ACSL3*. “The methylation of this gene is likely to regulate its expression, so it was therefore a good candidate for assessing a biological response to PAH exposure,” explains study coleader Frederica Perera, director of the Columbia Center for Children’s Environmental Health.

The researchers then confirmed their findings in a group of 56 women and their fetuses from the same cohort study. Of the children born to mothers with high PAH exposure, 81% had a methylated *ACSL3* gene promoter, compared with 23% of children born to mothers with low PAH exposure. An association was also found between the fetal methylation of *ACSL3* and parental reporting of asthma before the child’s fifth birthday, with 73% of asthmatic children showing *ACSL3* methylation compared with 41% of children without asthma. These findings were reported in the 16 February 2009 issue of *PLoS ONE*.

“This study is interesting but still preliminary,” remarks Manel Esteller, director of the Cancer Epigenetics and Biology Program at the Bellvitge Institute for Biomedical Research, Barcelona, Spain. “Results now need to be obtained with more subjects, the results duplicated in cell culture and animal models, and a mechanistic connection between PAH and the methylation of this gene demonstrated.”

Peter Helms, a professor of child health at the University of Aberdeen, United Kingdom, adds that the *ACSL3* link with asthma needs to be more convincingly demonstrated, with better control of possible confounding factors (such as maternal smoking), rigorous diagnostic criteria, and long-term follow-up. “Asthma is notoriously difficult to diagnose in children under age five, and if the study sample were larger and the children followed up for longer, I think we’d be closer to knowing whether methylation of *ACSL3* has potential in the early identification of childhood asthma,” he says.

Perera says the study authors plan to further test the association as the children mature and reach ages where they can be clinically diagnosed with asthma. If substantiated by future research, the methylation of *ACSL3* might serve as a tool to identify children at increased risk of developing asthma, thus opening up new avenues for prevention.

## Figures and Tables

**Figure:**
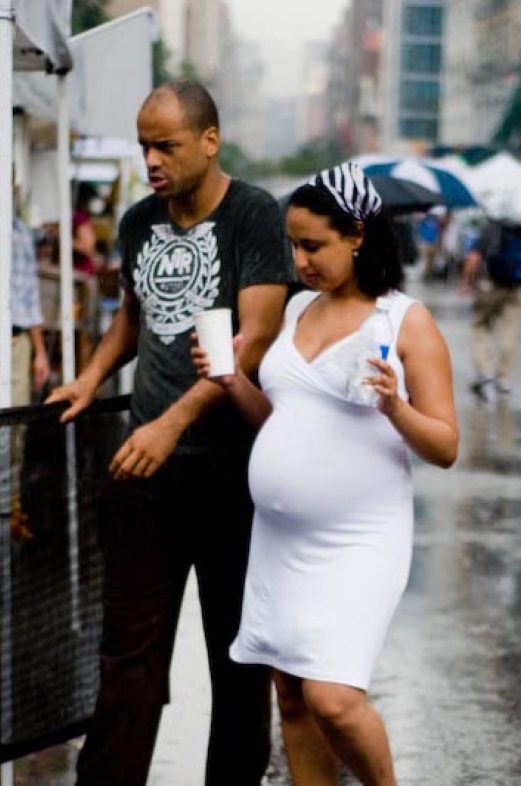
A study of pregnant black and Dominican women has revealed a potential biomarker predicting childhood asthma.

